# Comparative study of femtosecond laser ablation of glass using ultrathin gold and nickel films as sensitizers

**DOI:** 10.1038/s41598-026-67560-4

**Published:** 2026-09-14

**Authors:** Arpit Dave, Emmanuel Sarpong, Sabeeh Irfan Ahmad, Hsin-Yu Yao, Tsing-Hua Her

**Affiliations:** 1 Department of Physics and Optical Science, The University of North Carolina at Charlotte, 9201 University City Boulevard, Charlotte, NC 28223 USA; 2 Department of Physics, National Chung Cheng University, No.168, Sec.1, University Rd, Minhsiung, Chiayi 621301 Taiwan

**Keywords:** Materials science, Nanoscience and technology, Optics and photonics, Physics

## Abstract

It is known that an ultrathin Au film can sensitize femtosecond ablation of dielectric substrates by reducing the ablation threshold reduction and enhancing the ablation rate. In this work, we report the first observation that an ultrathin Ni film can also serve as a sensitizer for glass, although it is not as effective as Au. A comparative study of Au and Ni sensitizers provides clear evidence that metal-sensitized ablation of dielectric substrates is mediated by hot electrons in the metal film through interfacial electron-phonon coupling rather than phonon-phonon coupling. To understand the contrasting behaviors of these two metals, we present a simple thermodynamic model that examines the energy partitioning of hot electrons in metals between interfacial phonons and bulk phonons. We show that the relative strength of these energy-transfer pathways, rather than the absolute strength of the individual processes, plays a key role in determining the effectiveness of metal sensitizers. This relative strength is greater for Au than for Ni, making Au a superior sensitizer for optically induced metal-assisted dielectric breakdown.

**Supplementary Information:**

The online version contains supplementary material available at https://doi.org/10.1038/s41598-026-67560-4.

## Introduction

Optically induced metal breakdown (OMB) and optically induced dielectric breakdown (ODB) initiated by lasers have been studied extensively for many decades and have enabled many scientific, industrial, and defense applications^1,2^. For nanosecond laser ablation of wide-bandgap dielectric substrates where the one-photon absorption is weak, a variant was introduced in the early 2000s in which a thin metal film is directly deposited onto the dielectric substrate as a sensitizer to enhance light absorption. A reduction of the breakdown threshold ($\:{F}_{th}$) and an enhancement of the material removal rate (*MRR*) of dielectrics were demonstrated^3,4^. We dub this process optically induced metal-assisted dielectric breakdown (OMDB). Nanosecond OMDB has been observed for different metal films^5^, film thicknesses^5^, dielectric substrates^3^, laser wavelengths^3,4,6^, laser pulse numbers^5^, and laser spot sizes^5^. Several groups have qualitatively attributed this enhancement to a thermal process in which phonons in the metal film are strongly excited to very high temperatures, followed by heat transfer to the dielectric substrate via interfacial phonon-phonon coupling between the metal film and the substrate^3,4,7^. COMSOL modeling of the temperature distribution in an Au film sensitizer on sapphire was performed in^3^, but its accuracy is questionable because the optical and thermal properties are known to be strongly temperature dependent.

Meanwhile, there have been extensive studies of ultrashort laser interaction with metallic thin films on dielectric substrates on picosecond^8^ and femtosecond^9–11^ timescales. Pump-probe studies using NIR^9^, XUV^11^, and x-ray^10^ probe wavelengths have revealed non-thermal energy transport across metal-dielectric interfaces. This is corroborated by theoretical studies of interfacial thermal boundary resistance, which account for non-equilibrium electron and phonon populations in metals using a two-temperature model and underscore the role of direct coupling between electrons in the metal and phonons in dielectric substrates^12,13^. References^8–11^ employed low pulse energies such that the temperature rise in the dielectric substrates was negligible, and the substrate remained intact. In 2020, Wen et al. used high pulse energies and reported the first observation that a 12 nm Au film improved femtosecond laser ablation of sapphire, yielding cleaner ablation morphologies, lower ablation threshold fluences $\:{F}_{th}$, and higher material removal rates (*MRRs)*, than femtosecond ODB alone^14^. They attributed these enhancements to coupling between non-equilibrium hot electrons in the metal and phonons in the substrate, but this conjecture was neither proven experimentally nor validated theoretically. To date, this remains the only demonstration of femtosecond OMDB.

In this paper, we compare ultrathin Au and Ni films as sensitizers for femtosecond OMDB of glass. The motivation is as follows. While Au, as a noble metal, features predominantly s-band electrons near the Fermi level with ballistic electron transport, transition metals are known to possess a higher density of d-band electrons near the Fermi level and stronger electron-phonon coupling^15^. Noble metals therefore exhibit higher electron temperatures and lower phonon temperatures upon femtosecond laser heating than transition metals^16^. Here, we investigate this contrast to test the above conjecture^14^. Our findings demonstrate, for the first time, that an ultrathin Ni film can also improve femtosecond ablation of borosilicate glass, although its enhancement is less effective than that of Au. By comparing the ablation thresholds of these metal films with those of the corresponding metal-sensitized glass substrates, we conclude that femtosecond OMDB is mediated by direct interfacial coupling between electrons in the metal and phonons in dielectric substrates. This work therefore provides direct experimental validation of the conjecture proposed in^14^. We also introduce a simple thermodynamic model to explain why Au is a more effective sensitizer than Ni. Our theory provides physical insights into optimizing the efficacy of femtosecond OMDB.

## Experimental methods

The laser used in this study was a Coherent RegA 9000 Ti: Sapphire regenerative amplifier seeded by a Coherent Vitara-S oscillator, delivering ~ 120 fs pulses at 800 nm and 300 Hz. A single pulse was selected using a mechanical shutter. The pulse energy was controlled by a variable ND filter. A Parco 50$\:\times\:$ (0.55 NA) objective was used to focus the laser to a spot size of ~ 1.5 μm (full width at half maximum). Two samples, consisting of a 12.5-nm-thick Au film and a ~ 13.5-nm-thick Ni film sputtered onto separate borosilicate glass substrates, were investigated. These film thicknesses are comparable to the optical skin depth (extinction coefficient ≈ 4.91 for Au and ≈ 4.89 for Ni at 800 nm) and are smaller than the hot-electron diffusion lengths (~ 443 nm for Au and 31 nm for Ni^16^. Therefore, the electrons in both films can be considered uniformly heated. The sample was positioned relative to the focused laser beam using a three-axis Aerotech ANT-50 L stage. At each fluence, five exposures were performed to account for uncertainties in the pulse energy and in the area, depth, and volume of the ablated holes. The ablated regions were characterized using atomic force microscopy (AFM), and the resulting AFM images were analyzed using ImageJ and Gwyddion. All experiments and measurements were performed in air at room temperature.

## Results

**Fig. 1:**
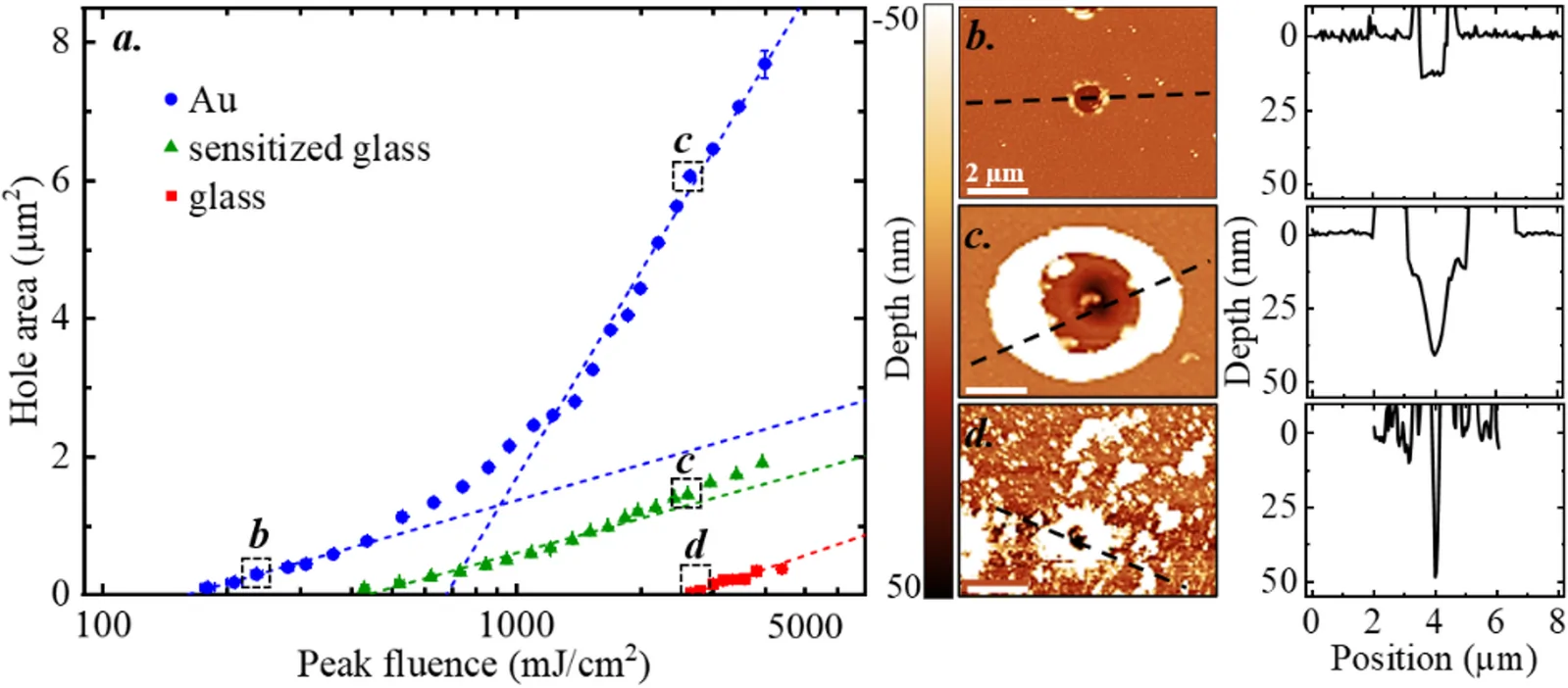
(**a**) Semi-log plot of the ablated hole area as a function of peak laser fluence for a ~ 12.5 nm Au film, Au-sensitized glass, and bare glass substrate. The dashed lines represent the corresponding least-squares fits according to^17^. All vertical error bars, except for the last point in the Au film data (± 0.2 µm^2^), are less than ± 0.06 µm^2^ and are smaller than the size of the data markers. (**b**-**d**). AFM images and their corresponding line profiles at the selected fluences indicated in (**a**) are displayed in Fig. 1b (Au/glass, 236 ± 8 mJ/cm²), *c* (Au/glass, 2600 ± 11 mJ/cm²), and *d* (bare glass, 2784 ± 10 mJ/cm²), respectively. Scale bars: 2 μm. See text for more details.

Figure 1a displays a semi-log plot of the ablated hole area as a function of the peak laser fluence, known as a Liu plot^17^, for a ~ 12.5 nm Au film (filled blue circles), Au-sensitized glass (filled green triangles), and bare glass (filled red squares). All error bars (except for the last point in the Au film data) in Fig. 1a are smaller than the size of the data markers. The dashed lines, which are linear fits to the data in the low-fluence regime and whose slopes correspond to the focused laser area according to^17^, exhibit similar slopes as expected. An increased slope is observed in the high-fluence regime, indicating the onset of a strong-ablation regime, which has previously been attributed to the long hot-electron diffusion length of ~ 443 nm in Au^16,18,19^. The extracted ablation thresholds for the Au film, Au-sensitized glass, and bare glass are $\:{F}_{th}^{Au}$ = 158 ± 7, $\:{F}_{th}^{glass-Au}$ = 431 ± 31, and $\:{F}_{th}^{glass}$ = 2596 ± 33 mJ/cm^2^, respectively. A 6$\:\times\:$ reduction in the $\:{F}_{th}$ of glass by the Au sensitizer $\left( { = F_{{th}}^{s} /F_{{th}}^{{s - Au}} } \right)$ is clearly observed.

AFM images and their corresponding line profiles at selected fluences in Fig. 1a are displayed in Fig. 1b, c, and *d*. Figure 1b shows clean removal of the Au film without substrate damage at 236 mJ/cm², while Fig. 1c shows a larger film removal accompanied by a dip into the glass substrate at 2600 mJ/cm². Both removed Au films are surrounded by protrusions resulted from re-solidification of molten Au, which is commonly observed in laser melting of this material^20^. The dip in the glass has a diameter of 1361 ± 1 nm and a depth of 29.5 ± 1.8 nm, yielding an ablated volume of (43 ± 0.3) ×10^− 3^ µm^3^. At a comparable fluence (Fig. 1d), the bare glass exhibits a dip with a diameter of 272 ± 7 nm and a depth of 50.0 ± 5.8 nm, yielding an ablated volume of (3 ± 0.4) ×10^− 3^ µm^3^. A ~ 14$\:\times\:$ increase in the *MRR* of glass by the Au sensitizer near the bare-glass ablation threshold is clearly observed.

**Fig. 2:**
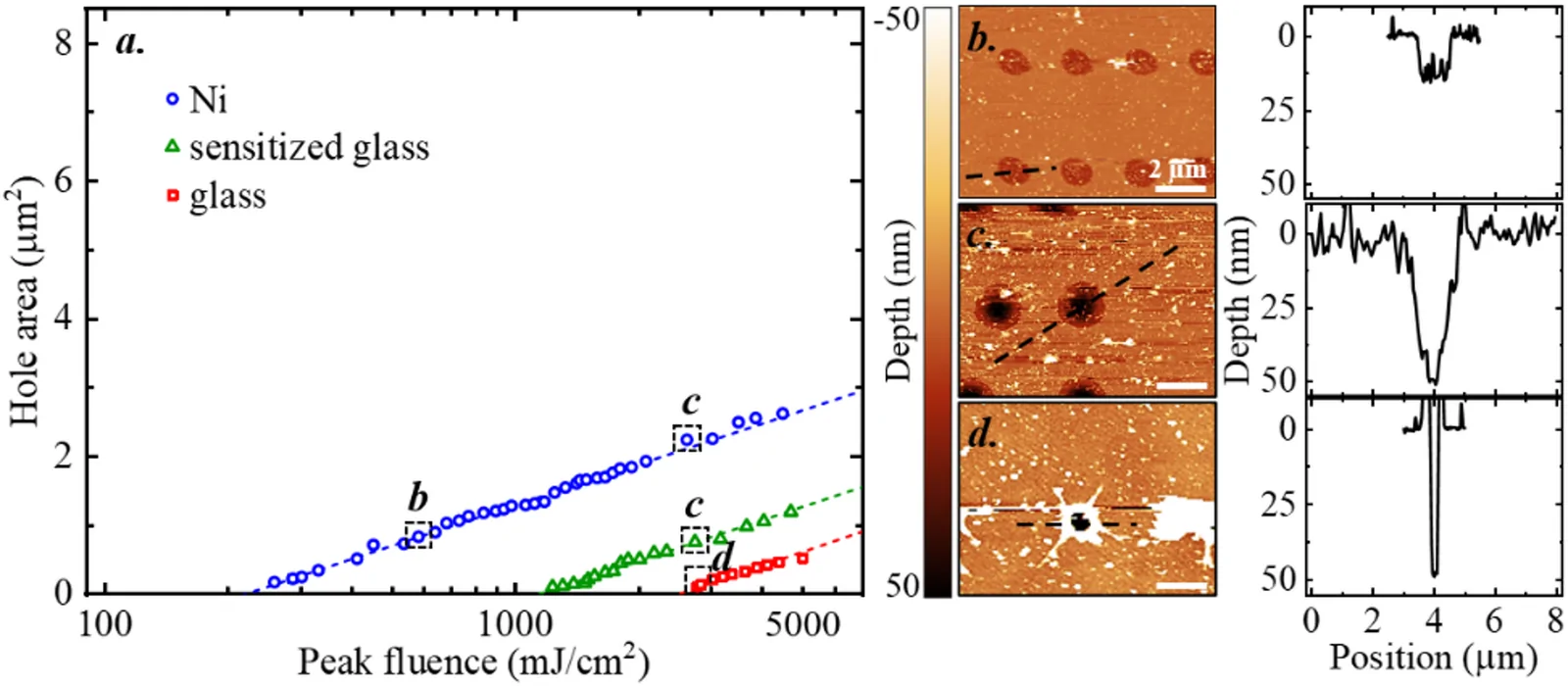
(**a**) Semi-log plot of the ablated hole area as a function of peak laser fluence for a ~ 13.5 nm Ni film, Ni-sensitized glass, and bare glass substrate. The dashed lines represent the corresponding least-squares fits according to^17^. The vertical error bars are less than ± 0.06 µm^2^ and are smaller than the size of the data markers. (**b-d**). AFM images and their corresponding line profiles at the selected fluences indicated in (**a**) are displayed in Fig. 2b (Ni/glass, 581 ± 8 mJ/cm²), *c* (Ni/glass, 2738 ± 8 mJ/cm²), *d* (bare glass, 2778 ± 10 mJ/cm²), respectively. Scale bars: 2 μm. See text for more details.

Figure 2a displays a semi-log plot of the ablated hole area as a function of peak laser fluence for a ~ 13.5 nm Ni film (hollow blue circles), Ni-sensitized glass (hollow green triangles), and bare glass (hollow red squares). The error bars in Fig. 2a are smaller than the size of the data markers. The dashed lines, which are linear fits to the data in the low-fluence regime and whose slopes correspond to the focused laser area according to^17^, exhibit similar slopes, as expected. The extracted ablation thresholds for the Ni film, Ni-sensitized glass, and bare glass are $\:{F}_{th}^{Ni}$ = 219 ± 5, $\:{F}_{th}^{glass-Ni}$ = 1134 ± 10, and $\:{F}_{th}^{glass}$ = 2485 ± 20 mJ/cm^2^, respectively. A 2.2$\:\times\:$ reduction in $\:{F}_{th}$ of glass by the Ni sensitizer $\left( { = F_{{th}}^{s} /F_{{th}}^{{s - Ni}} } \right)$ is clearly observed. We note that the ablation thresholds for the two bare glass substrates are consistent within ± 5%, indicating the reproducibility of our experiments.

As in the Au case, AFM images and their corresponding line profiles at selected fluences in Fig. 2a are shown in Fig. 2b, c, and *d*, respectively. Figure 2b shows clean Ni film removal without substrate damage, while Fig. 2c shows a larger film removal accompanied by a dip into the glass substrate. Both ablated Ni films exhibit much cleaner edges than the Au films, consistent with the much shorter hot-electron diffusion length of Ni (~ 31 nm) than that of Au (~ 443 nm)^16^. The dip in the glass has a diameter of 983 ± 13 nm and a depth of 36.5 ± 1.6 nm, yielding an ablated volume of (28 ± 1) ×10^− 3^ µm^3^. At a comparable fluence (Fig. 2d), the bare glass exhibits a hole with a diameter of 350 ± 2 nm and a depth of 50 ± 5 nm, yielding a volume of (4.8 ± 0.5) ×10^− 3^ µm^3^. A ~ 5.6$\:\times\:$ increase in the *MRR* of glass by the Ni sensitizer near the bare-glass ablation threshold is observed. This is the first demonstration that an ultrathin Ni film can sensitize femtosecond laser ablation of dielectric substrates, albeit less efficiently than an ultrathin Au film. This result shows that femtosecond metal-sensitized dielectric breakdown (fs-OMDB) is not limited to noble metals like Au but also applies to transition metals such as Ni.

**Fig. 3:**
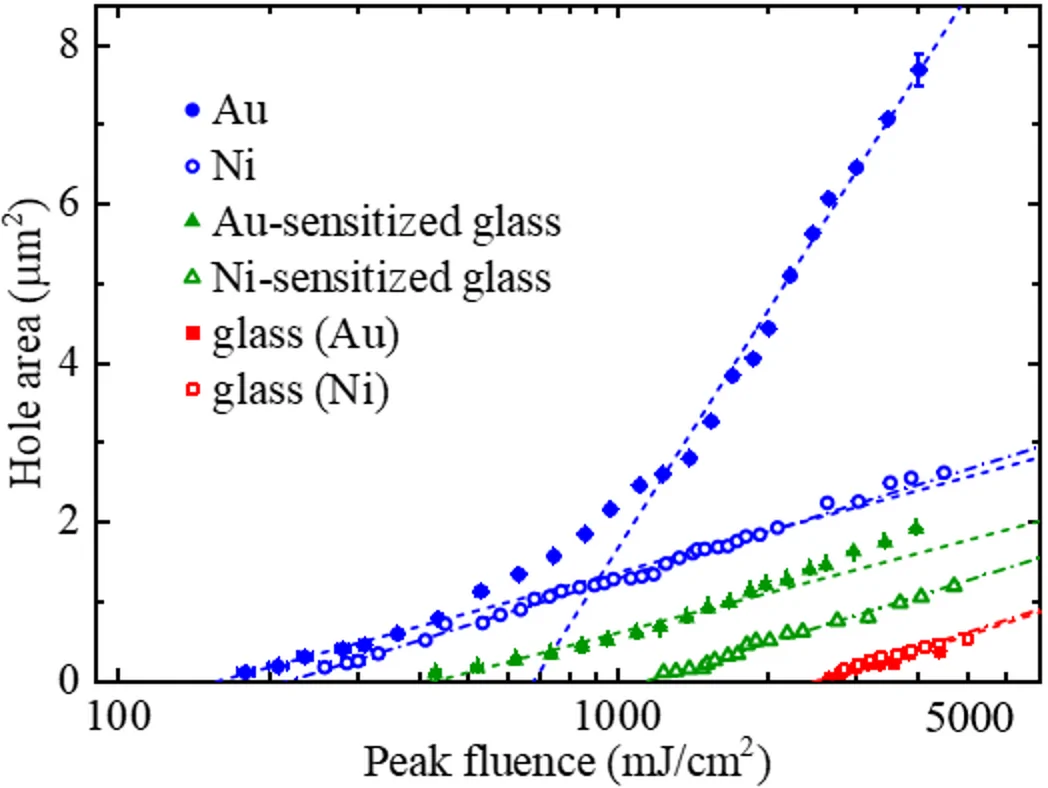
Combined plot of Fig. 1a (filled symbols) and Fig. 2a (hollow symbols) to facilitate direct comparison. Note that the filled and hollow red symbols nearly overlap, demonstrating the reproducibility of our experiments.

For direct comparison, Fig. 1a (filled symbols) and Fig. 2a (hollow symbols) are re-plotted in Fig. 3. It clearly shows that both Au and Ni can sensitize femtosecond ablation of glass, albeit with markedly different effectiveness. One straightforward measure is the ratio of $\:{F}_{th}^{glass}$ and $\:{F}_{th}^{glass-m}$, which is 6$\:\times\:$ for Au and 2.2$\:\times\:$ for Ni, indicating that Au is 2.7$\:\times\:$ more effective than Ni in sensitizing glass. From a theoretical perspective, however, it is more convenient to look at the ratio $\:{\xi\:}_{m}={F}_{th}^{s-m}/{F}_{th}^{m}$, which represents the multiple of $\:{F}_{th}^{m}$ required for glass ODB, once the OMB of the metal sensitizer is reached. For Au-sensitized glass, $\:{\xi\:}_{Au}=2.73\:\pm\:\:0.23$, whereas for Ni-sensitized glass, $\:{\xi\:}_{Ni}=5.18\:\pm\:\:0.13$. To quantify this contrast, we define a dimensionless effectiveness parameter $\:r={\xi\:}_{Ni}/{\xi\:}_{Au}=1.90\:\pm\:\:0.17\:$, which again indicates that Au is approximately 1.9$\:\times\:$ more effective than Ni at sensitizing ablation of glass once the metal sensitizer has reached its vaporization temperature. These quantities will be examined theoretically in the following discussion.

## Discussion

Figure 3 shows that fs-OMDB of these two metals is qualitatively similar but quantitatively very different. To understand this contrast, in the following we consider three highly non-equilibrium carriers generated by femtosecond laser heating of an ultrathin metal film on a semi-infinite dielectric substrate. Upon femtosecond laser excitation, electrons in the metal film are directly heated through photon absorption, resulting in a very high electron temperature, on the order of a few 1,000 K to a few 10,000 K depending on metals and fluence^16,21^, by the end of the laser pulse^22^. The phonons in the metal film are subsequently heated by these hot electrons via bulk electron-phonon coupling. Both the hot electrons and phonons can then transfer energy to phonons in the substrate through interfacial electron-phonon coupling and interfacial phonon-phonon coupling, respectively. We can readily rule out the latter as follows. It is well known that transition metals exhibit stronger bulk electron-phonon coupling than noble metals because of their higher electronic density of states near Fermi level^15^. As a result, for the same pulse energy, Au exhibits much hotter electrons and cooler phonons than Ni (see, for example Fig. 4 in^16^). Since our data show that $\:{F}_{th}^{glass-Au}$ is lower than $\:{F}_{th}^{glass-Ni}$ by a factor of 2.63, this indicates that electrons in the metal are more effective than phonons in transferring energy to the underlying substrate. Our comparative study of Au and Ni therefore provides clear evidence that interfacial electron-phonon coupling is the dominant mechanism responsible for fs-OMDB.

Having established that interfacial electron-phonon coupling is responsible for fs-OMDB, we next consider how this mechanism quantitatively explains the different sensitization efficiencies of Au and Ni. To quantitatively understand the interplay among the non-equilibrium electron temperature $\:{T}_{e}$, the phonon temperature, $\:{T}_{p}$, in the metal film, and the phonon temperature, $\:{T}_{s}$, in the dielectric substrate, it is necessary to solve a three-temperature model (3TM)^9^:

1 $$ \begin{aligned} C_{e} \frac{{dT_{e} }}{{dt}}\, \approx & \,\nabla \:\left( {\kappa \:_{e} \nabla \:T_{e} } \right) - G_{{ep,b}} \left( {T_{e} - T_{p} } \right) - \frac{{G_{{ep,i}} }}{d}\left( {T_{e} - T_{s} } \right) + S \\ C_{p} \frac{{dT_{p} }}{{dt}}\, \approx & \,G_{{ep,b}} \left( {T_{e} - T_{p} } \right) \\ C_{s} \frac{{dT_{s} }}{{dt}}\, \approx & \,\frac{{G_{{ep,i}} }}{d}\left( {T_{e} - T_{s} } \right) \\ \end{aligned}$$

where $\:{C}_{i}$ is the volumetric heat capacity (* i* = metal electron * e*, metal phonon * p*, or substrate phonon * s*), $\:{\kappa\:}_{e}$ is the electron thermal conductivity, $\:{G}_{ep,b}$ is the electron-phonon thermal conductance in the bulk region of the metal, $\:{G}_{ep,i}$ is the electron-phonon thermal conductance across the interface between the metal and the substrate (also known as electronic Kapitza conductance^23^), * d* is the metal film thickness, and * S* is the absorbed laser energy density. Equation (1) is challenging to solve for several reasons. First, even though $\:{C}_{e}\left({T}_{e}\right)$ and $\:{G}_{ep,b}\left({T}_{e}\right)$ for Au and Ni are known^15^, the values of $\:{G}_{ep,i}\left({T}_{e}\right)$ for these two metals and the glass substrate are, to the best of our knowledge, unknown. Second, the literature has pointed out that, under intense fs heating of metals, the latent heats of melting and evaporation must be included to account for phase transitions and phase explosion^21,24^. The 3TM would therefore need to be substantially modified to account for these phenomena, and many relevant thermal parameters are not known for glass. In the following, we propose a simple energy-partition model to explain $\:r=\frac{{\xi\:}_{Ni}}{{\xi\:}_{Au}}\sim1.9$.

The key behind this model is to consider how the input laser energy is distributed among different processes: bulk phonon heating, interfacial phonon heating, and lateral thermal diffusion. Lateral thermal diffusion can be neglected because the transverse thermal diffusion time due to hot electrons $\:\frac{{L}^{2}}{\left(\frac{{\kappa\:}_{e}}{{C}_{e}}\right)}$^25^ (~ 310 ps for Au and ~ 110 ps for Ni), is approximately one order of magnitude longer than the electron-phonon cooling time $\:\frac{{C}_{e}}{{G}_{ep,b}}$^26^ (~ 11.5 ps for Au and ~ 12.9 ps for Ni). Under this approximation, at the OMB threshold of the metal * m* ($\:m\:$= Au or Ni), the energy transferred to phonons in the metal film must equal its enthalpy $\:\varDelta\:{h}_{OMB}^{m}$ required for ablation. We therefore obtain the following energy-balance expression,

2 $$ \:u_{1}^{m} \cdot \:A_{1}^{m} \cdot \:a^{m} = \Delta \:h_{{OMB}}^{m} , $$

where $\:{u}_{1}^{m}$ is the incident pulse energy, $\:{A}_{1}^{m}$ is the absorbance by the electrons in metal * m*, and $\:{a}^{m}$ is the fraction of the absorbed energy in electrons coupled to phonons in the metal, all evaluated at the metal OMB threshold. Similarly, at the ODB threshold of the glass substrate, the energy transferred to phonons in the substrate must equal its enthalpy $\:\varDelta\:{h}_{ODB}^{s}$ required for ablation. We can therefore obtain the following energy balance expression,

3 $$ \:u_{2}^{m} \cdot \:A_{2}^{m} \cdot \:b^{m} = \Delta \:h_{{ODB}}^{s} ,$$

where $\:{u}_{2}^{m}$ is the incident pulse energy, $\:{A}_{2}^{m}$ is the absorbance by the electrons in metal * m*, and $\:{b}^{m}$ is the fraction of the absorbed energy in electrons coupled to substrate phonons, all evaluated at the glass ODB threshold. As shown in the Supplementary Information, $\:{A}_{1}^{m}\sim{A}_{2}^{m}$ within $\:\pm\:10\%$ over the electron-temperature range from $\:{10}^{4}K$ to $\:{3\times\:10}^{4}\:K$, which encompasses the temperatures relevant to common metal ablation and beyond^21,24^. Taking the ratio of Eqs. (3) and (2) yields

4 $$ \xi \:_{m} \cdot \:\frac{{b^{m} }}{{a^{m} }} = \frac{{\Delta \:h_{{ODB}}^{s} }}{{\Delta \:h_{{OMB}}^{m} }},$$

where $\:{\xi\:}_{m}=\frac{{u}_{2}^{m}}{{u}_{1}^{m}}=\frac{{F}_{th}^{s-m}}{{F}_{th}^{m}}.$ We note that $\:\varDelta\:{h}_{ODB}^{s}$ is a thermodynamic property of the substrate and is therefore independent of the metal sensitizer. Taking the ratio of Eq. (4) for Ni and Au to eliminate $\:\varDelta\:{h}_{ODB}^{s}$, and rearranging the resulting expression yields the effectiveness parameter

5 $$ r = \frac{{\xi \:_{{Ni}} }}{{\xi \:_{{Au}} }} = \frac{{\Lambda \:^{{Au}} \cdot \:\Delta \:h_{{OMB}}^{{Au}} }}{{\Lambda \:^{{Ni}} \cdot \:\Delta \:h_{{OMB}}^{{Ni}} }},$$

where $\:{\varLambda\:}^{m}=\frac{{b}^{m}}{{a}^{m}}$ is the branching ratio of the absorbed electron energy partitioned between interfacial and bulk phonons, reflecting the competition between interfacial and bulk electron-phonon coupling. We note that Eq. (5) is independent of the substrate, eliminating uncertainties associated with the substrate ODB and allowing us to focus on the role of metal sensitizer in fs-OMDB.

$\:{\varLambda\:}^{m}$ can be estimated as follows. For a given metal *m* subjected to femtosecond laser heating at time zero, we can approximate $\:\varLambda\:$, according to Eq. (1), as

6 $$\:\varLambda\:=\frac{b}{a}\approx\:\frac{{\int\:}_{0}^{\infty\:}\frac{{G}_{ep,i}\left({T}_{e}\right)}{d}\left({T}_{e}-{T}_{s}\right)dt}{{\int\:}_{0}^{\infty\:}{G}_{ep,b}\left({T}_{e}\right)\left({T}_{e}-{T}_{p}\right)dt}.$$

According to the theory of electron-phonon coupling^27^, $\:{G}_{ep,i}\left({T}_{e}\right)$ and $\:{G}_{ep,b}\left({T}_{e}\right)$ depend on the occupation numbers of the interfacial and bulk phonon modes, respectively. Since the phonon occupation number is governed by the Bose-Einstein distribution function, $\:{G}_{ep,i}\left({T}_{e}\right)$ and $\:{G}_{ep,b}\left({T}_{e}\right)$ share the same temperature dependence. It can be shown that $\:{G}_{ep,b}\left({T}_{e}\right)$ can be written as $\:{G}_{ep,b}\left({T}_{e}\right)=\:{g}_{b}\cdot\:f\left({T}_{e}\right)$, where $\:{g}_{b}=\frac{\pi\:\hslash\:{k}_{B}}{g\left({\epsilon\:}_{F}\right)}2{\int\:}_{0}^{\infty\:}\varOmega\:{\cdot\:\alpha\:}^{2}F\left(\varOmega\:\right)\cdot\:d\varOmega\:$ is temperature independent and $\:f\left({T}_{e}\right)={\int\:}_{-\infty\:}^{\infty\:}{g}^{2}\left(\epsilon\:\right)\cdot\:\left(-\frac{\partial\:{f}_{FD}}{\partial\:\epsilon\:}\right)d\epsilon\:$ is temperature dependent. In the above expressions, $\:{f}_{FD}$ is the Fermi Dirac distribution function, $\:{\alpha\:}^{2}F\left(\varOmega\:\right)$ is the electron-phonon spectral function with phonon frequency$\:\:{\Omega\:}$, $\:g\left(\epsilon\:\right)$ is the electron’s density of states of metal, and $\:{\epsilon\:}_{F}$ is its Fermi energy^15^. Similarly, $\:{G}_{ep,i}\left({T}_{e}\right)$ can be expressed as $\:{G}_{ep,i}\left({T}_{e}\right)=\:{g}_{i}\cdot\:f\left({T}_{e}\right)$, where $\:{g}_{i}$ has the same form as $\:{g}_{b}$ but with the electron-phonon spectral function corresponding to the interfacial phonon modes. Furthermore, because most of the energy transfer from electrons to phonons occurs when $\:{T}_{e}\:\gg\:{T}_{p}\:$and $\:{T}_{s}$, which is valid for times up to $\:{t}_{c}$ before electrons and phonons equilibrate (the exact choice of $\:{t}_{c}$ is not critical for our argument), $\:{T}_{p}\:$and $\:{T}_{s}$ can be neglected in Eq. (6), which then reduces to

7 $$\:\varLambda\:=\frac{b}{a}\approx\:\frac{{\int\:}_{0}^{{t}_{c}}\frac{{g}_{i}}{d}f\left({T}_{e}\right){T}_{e}dt}{{\int\:}_{0}^{{t}_{c}}{g}_{b}f\left({T}_{e}\right){T}_{e}dt}=\frac{{g}_{i}}{{g}_{b}d}.$$

This result indicates that the partitioning of electron energy between interfacial and bulk phonons is approximately temperature independent and is therefore applicable to both OMB of the metal and ODB of the glass. Using Eq. (7) and the fact that the Au and Ni films have nearly identical thicknesses in this study (12.5 and 13.5 nm, respectively), Eq. (5) can be rewritten as

8 $$\:r\approx\:\frac{{g}_{i}^{Au}}{{g}_{b}^{Au}}\frac{{g}_{b}^{Ni}}{{g}_{i}^{Ni}}\frac{\varDelta\:{h}_{OMB}^{Au}}{\varDelta\:{h}_{OMB}^{Ni}}=\frac{{G}_{ep,i}^{Au}\left({T}_{RT}\right)}{{G}_{ep,i}^{Ni}\left({T}_{RT}\right)}\frac{{G}_{ep,b}^{Ni}\left({T}_{RT}\right)}{{G}_{ep,b}^{Au}\left({T}_{RT}\right)}\frac{\varDelta\:{h}_{OMB}^{Au}}{\varDelta\:{h}_{OMB}^{Ni}}.$$

The last equality follows from the fact that $\:\frac{{g}_{i}^{m}}{{g}_{b}^{m}}$ is temperature independent and can therefore be replaced by its room-temperature value, $\:\frac{{G}_{ep,i}^{m}\left({T}_{RT}\right)}{{G}_{ep,b}^{m}\left({T}_{RT}\right)}$. The values of $\:{G}_{ep,b}$ at room temperature are listed in Table 1. Unfortunately, to the best of our knowledge, values of $\:{G}_{ep,i}$ for Au-glass and Ni-glass are not available in literature. On the other hand, the literature reports the following room temperature values (in units of $\:MW{m}^{-2}{K}^{-1}$): $\:\frac{{G}_{ep,i}^{Au}}{{G}_{ep,i}^{Ti}}=\frac{350\:}{3000}\approx\:0.12$ for SiO_2_ substrates^28^, $\:\frac{{G}_{ep,i}^{Au}}{{G}_{ep,i}^{Ni}}=\frac{36\:}{155}\approx\:0.23$ for GaN substrates^29^, and $\:\frac{{G}_{ep,i}^{Au}}{{G}_{ep,i}^{Ni}}=\frac{71\:}{200}\approx\:0.36$ for Si substrates^30^. These results suggest that a value of $\:\frac{{G}_{ep,i}^{Au}}{{G}_{ep,i}^{Ni}}$ between 0.1 and 0.4 is reasonable. Combined this with $\:\frac{{{G}_{ep,b}}^{Ni}}{{{G}_{ep,b}}^{Au}}$ = 17.1 (from Table 1), gives $\:1.7<\frac{{\varLambda\:}^{Au}}{{\varLambda\:}^{Ni}}=\:\frac{{G}_{ep,i}^{Au}}{{G}_{ep,i}^{Ni}}\frac{{G}_{ep,b}^{Ni}}{{G}_{ep,b}^{Au}}<6.8$. Finally, for thermodynamic evaporation of metal *m*, we have $\:\varDelta\:{h}_{OMB}^{m}=\left({C}_{p}^{m}{\varDelta\:T}_{v}^{m}+\varDelta\:{H}_{v}^{m}\right)\cdot\:{d}^{m}$, where $\:\varDelta\:{H}_{v}^{m}$ is the volumetric enthalpy of evaporation and $\:{d}^{m}$ is the metal film thickness. Based on the values listed in Table 1, we obtain $\:\frac{\varDelta\:{h}_{OMB}^{Au}}{\varDelta\:{h}_{OMB}^{Ni}}$ $\:\approx\:$ 0.6. Combining these results yields $\:1\:<r\:<4$, which is within a factor of two of our experimental value of * r=1.9*. We acknowledge that femtosecond laser ablation is a complex process involving melting, heat transport in the liquid phase, vaporization, phase explosion, and hydrodynamic mass transport. Many of these processes have recently been incorporated into two-temperature models for femtosecond laser ablation of bulk metals^21,24^. Extending such models to a three-temperature framework for the laser intensities investigated here has not yet been demonstrated and is certainly beyond the scope of the present work.

**Table 1:** Material constants for calculations. Vaporization temperatures,$\:{T}_{v}$. are from^31^. Volumetric enthalpies of vaporization,$\:\varDelta\:{H}_{v}^{m}$, are converted from molar enthalpies from^31^ divided by molar densities. Volumetric phonon heat capacities,$\:{C}_{p}$, and bulk electron-phonon thermal conductance,$\:{G}_{ep,b}$, at room temperature are from^16^.

	$\:{T}_{v}\:\left(K\right)$	$\:{C}_{p}\:\left(\frac{J}{{m}^{3}K}\right)$	$\:\varDelta\:{H}_{v}^{m}\:\left(\frac{J}{{m}^{3}}\right)$	$\:{G}_{ep,b}\:\left(\frac{W}{{m}^{3}K}\right)$
Au	3243	$\:2.5\times\:{10}^{6}$	$\:3.4\times\:{10}^{10}$	$\:2.1\times\:{10}^{16}$
Ni	3003	$\:4.1\times\:{10}^{6}$	$\:5.7\times\:{10}^{10}$	$\:36\times\:{10}^{16}$

Our model developed above provides an important clue as to why Au is a more effective sensitizer than Ni (i.e., * r>1*). Although Ni exhibits stronger interfacial electron-phonon coupling than Au ($\:0.1<\:\frac{{G}_{ep,i}^{Au}}{{G}_{ep,i}^{Ni}}<0.4$), its bulk electron-phonon coupling is even stronger relative to that of Au ($\:\frac{{G}_{ep,b}^{Au}}{{G}_{ep,b}^{Ni}}=0.056$). As a result, $\:1.7<\frac{{\varLambda\:}^{Au}}{{\varLambda\:}^{Ni}}<6.8$, which is the key for the effective parameter * r>1*. Although our model is simplified, it highlights that the more favorable energy partitioning in Au plays a key role in its superior performance over Ni in femtosecond OMDB.

## Conclusion

We study femtosecond laser ablation of glass coated with ultrathin Au and Ni films and show that both metals act as sensitizers. As Au and Ni are representative of noble and transition metals, respectively, our findings strongly suggest that femtosecond OMDB is a universal phenomenon, although a comprehensive future study involving a wider range of metals is needed to validate this claim. Comparing Au and Ni sensitizers provides clear evidence that substrate ablation is mediated by hot electrons in the metal through interfacial electron-phonon coupling, thereby confirming an earlier conjecture^14^. We show that Au is a more effective sensitizer than Ni for fs-OMDB in three ways. First, the ablation threshold reduction is 6$\:\times\:$ for Au and 2.2$\:\times\:$ for Ni. Second, the enhanced material removal rate is enhanced by a factor of 14.3$\:\times\:$ for Au and 5.6$\:\times\:$ for Ni near the bare glass ablation threshold. Third, once the OMB threshold of the metal sensitizer is reached, the additional fluence required for glass ODB is 1.9$\:\times\:$ lower for Au than for Ni ($\:{r}_{\xi\:}=1.9$). To explain the latter, we present a thermodynamic model that examines the competition between interfacial and bulk electron-phonon coupling in each metal at its OMB and OMDB thresholds. We show that the relative strength of these competing coupling pathways, rather than the absolute strengths of individual processes, plays a key role in determining the effectiveness of metal sensitizers. This relative strength is greater for Au than for Ni, making Au a superior sensitizer for femtosecond OMDB.

## Supplementary Information

Below is the link to the electronic supplementary material.

Supplementary Material 1

## Data Availability

The data supporting the conclusions are contained within the manuscript and supplementary information. The raw data can be provided upon reasonable request.
