# Supplementary material for: Comparative study of femtosecond laser ablation of glass using ultrathin gold and nickel films as sensitizers
Source: Sci Rep. 2026 Sep 14;16:28610. doi: 10.1038/s41598-026-67560-4 (PMC13575177; doi:10.1038/s41598-026-67560-4)
Supplement: Supplementary file 1 [file 41598_2026_67560_MOESM1_ESM.pdf]

Comparative study of femtosecond laser ablation of glass using ultrathin gold and nickel films as sensitizers

Arpit Dave[1], Emmanuel Sarpong[1], Sabeeh Irfan Ahmad[1], Hsin-Yu Yao[2], and Tsing-Hua Her[1]

[1]Department of Physics and Optical Science, The University of North Carolina at Charlotte, 9201 University City Boulevard, Charlotte NC 28223, USA

[2]Department of Physics, National Chung Cheng University, No.168, Sec. 1, University Rd., Minhsiung, Chiayi 621301, Taiwan

Corresponding authors: Tsing-Hua Her, ther@charlotte.edu. Hsin-Yu Yao, hsinyuyao@ccu.edu.tw.

To estimate the change in optical absorptance during femtosecond laser heating of electrons, we adopted the Brendel-Bormann dielectric model introduced by Rakić *et al.* [1]. According to their Eq. (11), the complex dielectric function ε_m at laser frequency ω is

$$\varepsilon_m(\omega) = 1 - \frac{\Omega_p^2}{\omega(\omega - i\Gamma_0)} + \sum_{j=1}^{k} \chi_j(\omega), \tag{S1}$$

where the first two terms represent the plasma (free carrier) response with plasma frequency Ω_p and total electron collision frequency Γ_0, while the summation term represents the contribution of bound electrons $\chi_j(\omega)$ given in Eq. (10) of [1]. The relevant model parameters are taken from Tables 1 and 3 of [1].

To incorporate the effect of the non-equilibrium electron temperature, T_e, and the phonon temperature, T_p, arising from ultrafast femtosecond-laser heating of the metal film, Γ_0 in Eq. (S1) is replaced with the temperature-dependent total collision frequency [2],

$$\Gamma_0 = AT_e^2 + BT_p. \tag{S2}$$

The A and B coefficients for Au, as reported in [2], are 1.2×10^{7} Hz/K^2 and 1.23×10^{11} Hz/K, respectively. However, to the best of our knowledge, the corresponding values for Ni are not available in the literature, and are therefore estimated below. Since the electron thermal conductivity is related to the electron heat capacity through the Drude relation [3],

$$\kappa_e = v_F^2 C_e \tau_e / 3, \tag{S3}$$

where ν_F is the Fermi velocity, $C_e(T_e)$ is the volumetric electron heat capacity, and $\tau_e = 1/\Gamma_0$ is the total electron scattering time. Based on the electron thermal conductivity of Ni reported in [4], we use Eqs. (S2) and (S3) to extract the corresponding A and B coefficients, yielding values of 3.44×10^{7} Hz/K^2 and 4.6×10^{12} Hz/K, respectively.

To calculate the absorptance of ultrathin metal films, we model each film as an etalon consisting of air/metal film/glass. For a metal film of thickness L_m and index $n_m = \sqrt{\varepsilon_m}$, deposited on a glass substrate with a refractive index $n_s = 1.475$, the transmission and reflection coefficients of the etalon can be expressed as

$$t = \frac{t_L t'_R \exp(i k_m L_m)}{1 - r'_L r'_R \exp(2 i k_m L_m)}, \tag{S4}$$

$$r = r_L + \frac{t_L t'_L r'_R \exp(2 i k_m L_m)}{1 - r'_L r'_R \exp(2 i k_m L_m)}, \tag{S5}$$

where $k_m = n_m \, \omega / c$ is the propagation constant inside the metal film. The Fresnel coefficients $t_L = 2/(1 + n_m)$ and $r_L = (1 - n_m)/(1 + n_m)$ denote the transmission and reflection coefficients at the air-metal interface for light incident from air. Likewise, $t'_L = 2n_m/(n_m + 1)$ and $r'_L = (n_m - 1)/(n_m + 1)$ denote the corresponding coefficients at the metal-air interface for light incident from within the metal, while $t'_R = 2n_m/(n_m + n_s)$ and $r'_R = (n_m - n_s)/(n_m + n_s)$ denote the corresponding coefficients at the metal-glass interface for light incident from within the metal. The steady-state absorptance of the metal film can then be evaluated as

$$A = 1 - n_s |t|^2 - |r|^2. \tag{S6}$$

During the initial electron-heating stage under femtosecond laser irradiation (approximately the first $100 \sim 200$ fs), T_p, is assumed to remain fixed at 300 K. High electron temperatures, T_e, ranging from 10^4 K to 3×10^4 K are then considered to evaluate whether the absorptance

changes under conditions relevant to optically induced metal breakdown (OMB) and optically induced metal-sensitized dielectric breakdown (OMDB).

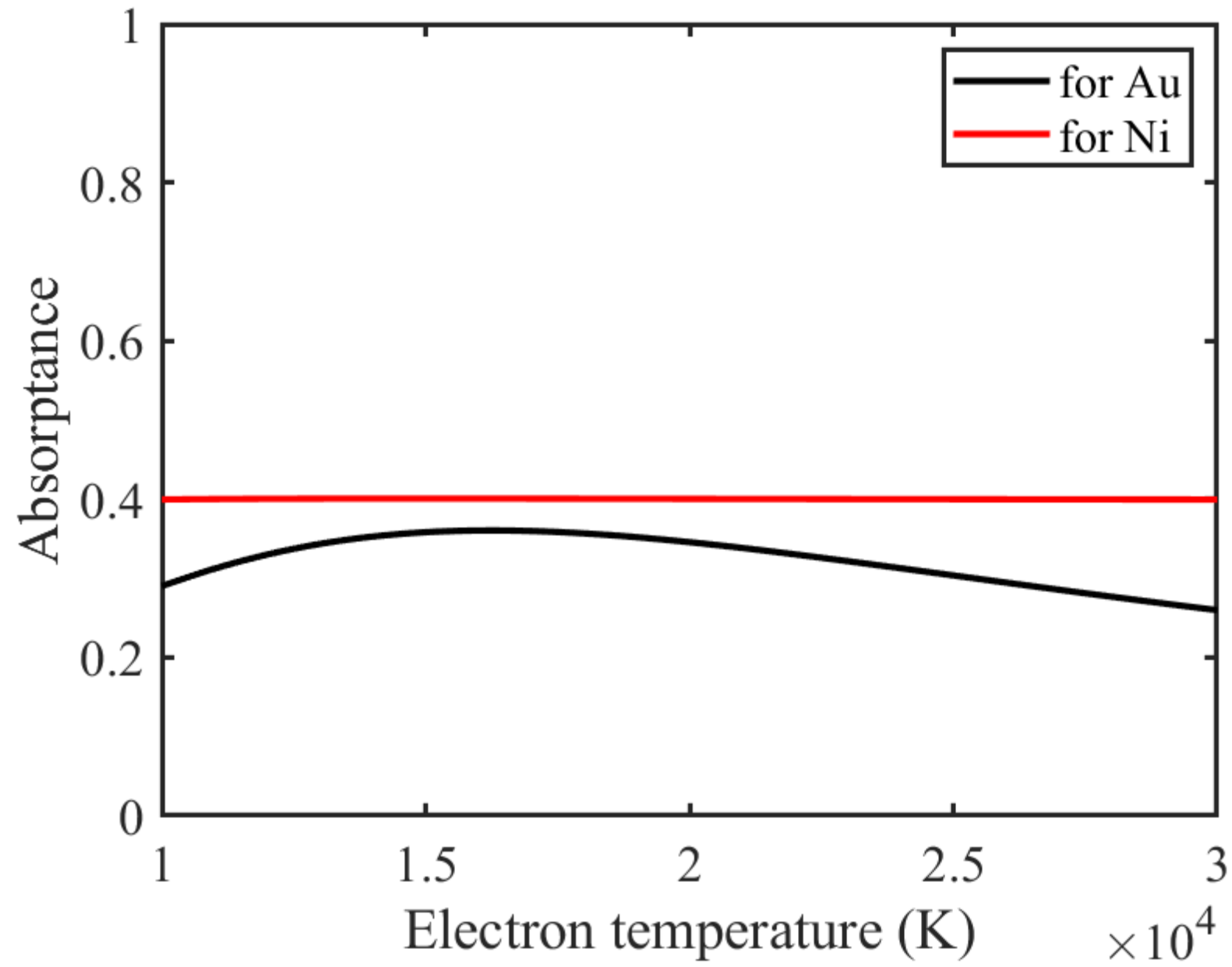

Fig. S1. Absorptance as a function of electron temperature for 12.5-nm-thick Au (black curve) and 13.5-nm-thick Ni (red curve) films.

Figure S1 clearly shows that the absorptance of Ni remains nearly constant as T_e increases from 10^4 K to 3×10^4 K. In contrast, the absorptance of the Au film exhibits a slight non-monotonic dependence on T_e. Nevertheless, the overall variation remains within $\pm 10\%$. Based on this observation, it is reasonable, to first order, to assume constant absorptance when estimating the deposited-energy ratio for OMB and OMDB in the main text.

References:

1. Rakić, A.D., et al., *Optical properties of metallic films for vertical-cavity optoelectronic devices.* Applied optics, 1998. **37**(22): p. 5271-5283.
2. Wang, X., et al., *Time-resolved electron-temperature measurement in a highly excited gold target using femtosecond thermionic emission.* Physical Review B, 1994. **50**(11): p. 8016.
3. Ashcroft, N.W. and N.D. Mermin, *Solid state physics*. 1976, holt, rinehart and winston, new york London.
4. Petrov, Y.V., et al. *Electron thermal conductivity of nickel and aluminum in solid and liquid phases in two-temperature states*. in *Journal of Physics: Conference Series*. 2021. IOP Publishing.
